# Affective Pictures and the Open Library of Affective Foods (OLAF): Tools to Investigate Emotions toward Food in Adults

**DOI:** 10.1371/journal.pone.0158991

**Published:** 2016-08-11

**Authors:** Laura Miccoli, Rafael Delgado, Pedro Guerra, Francesco Versace, Sonia Rodríguez-Ruiz, M. Carmen Fernández-Santaella

**Affiliations:** 1 The University of Granada Mind, Brain, and Behavior Research Center (CIMCYC), Granada, Spain; 2 The University of Oklahoma Stephenson Cancer Center, Department of Family & Preventive Medicine, Oklahoma Tobacco Research Center, Oklahoma City, Oklahoma, United States of America; University of Florida, UNITED STATES

## Abstract

Recently, several sets of standardized food pictures have been created, supplying both food images and their subjective evaluations. However, to date only the OLAF (Open Library of Affective Foods), a set of food images and ratings we developed in adolescents, has the specific purpose of studying emotions toward food. Moreover, some researchers have argued that food evaluations are not valid across individuals and groups, unless feelings toward food cues are compared with feelings toward intense experiences unrelated to food, that serve as benchmarks. Therefore the OLAF presented here, comprising a set of original food images and a group of standardized highly emotional pictures, is intended to provide valid between-group judgments in adults. Emotional images (erotica, mutilations, and neutrals from the International Affective Picture System/IAPS) additionally ensure that the affective ratings are consistent with emotion research. The OLAF depicts high-calorie sweet and savory foods and low-calorie fruits and vegetables, portraying foods within natural scenes matching the IAPS features. An adult sample evaluated both food and affective pictures in terms of pleasure, arousal, dominance, and food craving, following standardized affective rating procedures. The affective ratings for the emotional pictures corroborated previous findings, thus confirming the reliability of evaluations for the food images. Among the OLAF images, high-calorie sweet and savory foods elicited the greatest pleasure, although they elicited, as expected, less arousal than erotica. The observed patterns were consistent with research on emotions and confirmed the reliability of OLAF evaluations. The OLAF and affective pictures constitute a sound methodology to investigate emotions toward food within a wider motivational framework. The OLAF is freely accessible at digibug.ugr.es.

## Introduction

### Pictures in research on food cue reactivity

In research on human food cue processing, images of food are frequently employed to prompt reactions in the laboratory and researchers typically compare reactions to high-calorie food cues with reactions to low-calorie foods and/or non-food neutral objects [1–3]. Despite the widespread use of images in food cue processing research, only recently some groups of researchers developed standardized sets of visual food stimuli, like Food.Pics [4], Full4Health Image Collection/F4H [5], and the FoodCast Research Image Database/FRIDa [6]. In these digitized sets, food pictures are first edited and displayed against a uniform background, for a fine-grained control of perceptual features. Subsequently, wide samples of participants rate food and neutral non-food images on different scales. Therefore, standardized sets of digitized food pictures provide researchers worldwide with easy to access, edited food cues that have additionally been evaluated on dimensions related to subjective likability. The food cue reactivity paradigm, by far the most prevalent paradigm to investigate healthy and pathological reactions to food cues in the laboratory, assumes that certain individuals, assigning greater motivational relevance to high-calorie food stimuli, will show greater subjective, behavioral, and physiological reactivity to these food cues relative to other low in calories or non-food items [7]. However, some investigations have failed to observe psychophysiological differences between reactions to high-calorie and low-calorie food [8] and between clinical, namely, obese, and healthy participants [2], overall indicating the importance of reliably pinpointing the most motivationally relevant food stimuli. As a further matter, pivotal for the present investigation, some researchers argue [9] that when evaluating food cues one cannot assume that the values along the judgment scale make reference to the same feelings/sensations in different individuals, especially if obese, rendering pleasure /likability ratings for food cues unreliable across individuals and groups unless those ratings are standardized against ratings of extreme experiences unrelated to food. Accordingly, we propose here that highly emotional images, included as non-food control stimuli, can serve as affective terms of comparisons during the evaluation of food pictures, providing the extreme experiences unrelated to food that, based on Bartoshuk and colleagues' recommendations [9], allow drawing valid evaluations of food cues.

### Pictures in research on emotional cue processing

In affective neuroscience, standardized sets of natural images like the International Affective Picture System [10] and like the more recent EmoPics [11] have helped identifying reliable emotional reactions, visible through language, behavior, and physiology [12]. In the IAPS, affective images aim to represent 'the variety of human experience' and, accordingly, depict diverse content within natural scenes: babies, families and puppies, erotic couples, war scenes, threats and mutilations, landscapes, adventure and sport scenes, household objects… For each IAPS picture, wide samples of individuals provide “normative affective ratings” notably of pleasure (good/bad) and emotional arousal (dull/extremely emotional), thus pinpointing the subjective emotional value of each image. Afterwards, the normative ratings serve as a benchmark to identify patterns in the human physiology of emotion. Thus, images with extreme ratings in both pleasure and arousal (e.g., erotica, mutilations, personal threats) tend to trigger the clearest physiological reactions (for a review, see, e.g., [13]).

We put forward that data emerged in affective neuroscience using the “affective picture viewing” paradigm offer clues to unravel ambiguous results in human food cue processing, pointing out that food images elicit relatively weak emotional reactions, obtaining high ratings for pleasure but low ratings for arousal [13] and, crucially, prompting physiological reactions that are at times indistinguishable from those to images with neutral content [12]. Therefore, identifying the most appetitive food images, characterized by the highest ratings in both pleasure and arousal, has the potential of boosting the psychophysiological reactions to food cues, in turn helping to look into differences in food cues processing between obese or eating disordered and healthy individuals. Based on this line of reasoning, we advocate the inclusion of affective scenes as non-food control stimuli during food cue viewing, in the first place, as mentioned earlier, to reach the methodological goal of obtaining valid food picture evaluations. However, emotional cues [10,11,14] are typically presented within the context of natural settings that, from a perceptual point of view, differ considerably from the figure/ground lab friendly display of foods provided in the current digitized sets of food images [4–6]. The visual mismatch between figure/ground food images and affective pictures renders inadvisable to examine physiological variables, like early cortical components, that are influenced by the perceptual characteristics of the stimuli [15], consequently calling for the creation of food picture sets that display foods within natural scenes. In addition to these methodological considerations, the theoretical rationale for including emotional stimuli in food cue processing research is that they offer a wider framework to infer the emotional relevance of food as compared to other rewarding and non-rewarding stimuli, allowing to investigate whether in clinical populations the presence of altered emotional processing of food modifies the significance of motivationally relevant stimuli other than food [16].

### OLAF, the Open Library of Affective Foods

To the best of our knowledge, only a set of food pictures that we developed lately (OLAF, the Open Library of Affective Foods [17]) reported subjective evaluations of both affective and food images and presented foods within natural scenes, aimed to match the IAPS perceptual features. The first version of the OLAF specifically focused on an adolescent sample, describing the emotional processing of food cues during youth. During the evaluation of the OLAF, the diverse non-food images from the IAPS serve as emotional anchors, to guarantee that the participants grasp the full range of the scales they need to employ: valence, arousal, dominance, and food craving. For ethical reasons, in the adolescent version of the OLAF the non-food emotional pictures were age-appropriate and, accordingly, consisted only of less arousing contents from the IAPS, like adventure scenes, babies, and war scenes with guns not directed toward the viewer. In the present investigation, we report on the OLAF pictures and ratings from an adult population. The non-food emotional anchors from the IAPS consisted of the emotional categories that prompt some of the most extreme affective reactions: erotica, neutral objects, and mutilations, thus ensuring the reliability of food cues evaluations. To further secure the reliability of the ratings, both versions of the OLAF conformed to the standardized IAPS ratings procedures [10], that include 1) employing the Self-Assessment Manikin (SAM), a set of non-verbal pictorial scales to probe emotional reactions to pictures in different cultures [13,18]; 2) in the rating instructions, using several words to clarify the gist of each affective dimension (e.g., for arousal: "completely relaxed, calm, sluggish, dull, sleepy, unaroused" vs. "stimulated, excited, frenzied, jittery, wide-awake, aroused "); and 3) recruiting wide samples of individuals (> = 100/set) while also limiting the number of pictures to be rated (60 images/set). The use of non-standardized procedures to obtain affective ratings (e.g., [19,20]) has been associated with judgments that are inconsistent with the typical patterns [12] and do not differentiate pleasure from arousal.

Summarizing, we here provide: 1) the OLAF, a set of original food pictures that perceptually match IAPS images; and 2) affective ratings of pleasure, arousal, dominance, and food craving for the OLAF and IAPS pictures, following the standardized IAPS procedures [10].

Our ultimate goal is to advocate for the simultaneous use of food and emotional images to foster research on how eating disordered and obese individuals process food compared to other relevant emotional content, going “beyond the cue-reactivity paradigm” [16] to investigate the role of hedonic factors in eating and weight-related disorders [21].

The OLAF food images, affective ratings, and methods guidelines are fully accessible at the following link, that belongs to “digibug”, the University of Granada public repository. http://hdl.handle.net/10481/41499

## Materials and Methods

### Participants

The sample consisted of 424 students from the University of Granada who received course credit for their participation. Table 1 summarizes the participants' demographics. The male-female ratio of 1:1.71 was in line with those of previous studies collecting normative affective ratings [22,23]. Data were collected at two time points across a 10-month period (first time point, N = 341; second time point, N = 83). Due to computer failure, 8 participants (2 males) could not complete the questionnaires after evaluating the pictures. The University of Granada Institutional Review Board approved the study (IRB# 837). All participants provided written informed consent.

**Table 1:** Demographic characteristics of the sample.

Participants’ characteristics	N (%) or Mean (SD)	Overall N
**Age**	20.9 (4.1)	424
**Gender**		
Females	269 (65.44%)	424
Males	155 (36.56%)	
**Hunger PRE picture evaluation**		
“Yes, I am hungry”.	105 (24.76%)	424
“No, I am not hungry”.	319 (75.24%)	
**Body Mass Index**		
BMI < = 18	30 (7.08%)	424
18> BMI < = 25	311 (73.35%)	
25> BMI <30	68 (16.04%)	
BMI > = 30	15 (3.54%)	
**Social Desirability** ^a^	14.5 (4.5)	416
**Snaith-Hamilton Pleasure Scale** ^a^	46.8 (4.6)	416
**Food Craving-Trait** ^a^	110.7 (25.7)	416

^**a**^ Due to computer failure, 8 participants (2 males) could not complete the questionnaires after evaluating the pictures and are therefore not included in the table.

### Materials

The IAPS and OLAF pictures were displayed in 4 orders, randomized across rating sessions. Each picture order included 36 IAPS (the same for all orders, with 12 pictures per affective category) and 24 OLAF images (different for each order, with 6 pictures per food category), leading to a total of 60 images that were presented intermixed and without category repetitions. Each order began with a different category and was presented to at least 103 participants (106 on average).

Affective pictures from the IAPS served as the control stimuli and comprised pleasant (IAPS codes: 4290, 4311, 4659, 4664, 4668, 4670, 4695, 4810, 4693, 4697, 4698, 4800), unpleasant (IAPS codes: 3000, 3015, 3053, 3063, 3064, 3080, 3102, 3131, 3168, 3170, 3266, 9410), and neutral images (IAPS codes: 7000, 7006, 7010, 7041, 7150, 7175, 7185, 7217, 7491, 7705, 7950, 9360). Using normative ratings [10], we selected the most arousing IAPS contents, erotica and mutilations, and household objects as neutrals, so that the average pleasure ratings significantly differed across IAPS contents (erotica: 6.67 (1.9), neutrals: 4.81 (1.0), mutilations: 1.49 (1.0)) and so that the mean arousal ratings were the same for erotica and mutilations, while significantly greater than the IAPS ratings for neutral objects (erotica: 6.80 (1.9), neutrals: 2.37 (1.7), mutilations: 6.70 (2.3)).

The OLAF images included 96 original images, of homemade meals and restaurants, depicting both low-calorie (fruits and vegetables) and high-calorie (savory and sweet) foods, with 24 items per category, distributed across picture orders. Within each food category, 6 subtypes of food were identified, providing 4 exemplars of the same food subtype, one for each of the 4 picture orders. In-depth details on the development of the OLAF images can be found in a previous manuscript [17], in which adolescents evaluated OLAF pictures and IAPS images that were lower in pleasure and arousal. Adolescent OLAF ratings unmasked four emotionally “less effective” savory high-calorie foods with lower valence and arousal ratings (OLAF codes: fat_0012, fat_0042, fat_0611, fat_0721). Accordingly, these pictures were replaced in the adults set by new OLAF images of similar content (OLAF codes: fat_0021, fat_0025, fat_0123, fat_0224). The non-verbal Self-Assessment Manikin/SAM scales were employed to assess subjective feelings of pleasure, arousal, dominance (Bradley & Lang, 1994), and food craving [17,24]. The SAM craving scale was initially developed for tobacco cravings [24] and subsequently extended to food cravings [17]. It displays emotions along a pictorial continuum, likewise the classic dimensions of the Self-Assessment Manikin, and ranges from a face with a mouth shut to a face with a drooling mouth.

### Procedure

As many as 25 university students enrolled in each rating session, described as involving research on food and physical activity habits. The rating sessions were scheduled between 10 am and 5 pm in a Psychology Department computer laboratory that was kept at a constant dim light. After collecting informed consent, experimenters provided standardized instructions [10] on the use of SAM and the additional questionnaires. The SAM instructions and the OLAF/IAPS images were displayed onto a white screen (average picture size: 1.87 m, horizontal; 1.37 m, vertical) using an Epson EMP-54 projector. Stimuli delivery was controlled using Presentation (v.16.3, Neurobehavioral Systems, San Francisco, CA) on a Toshiba Satellite ProA120 laptop. To provide subjective evaluations, each participant used a HP Compaq dc7700 personal computer running EPrime v.2.0 [25] and sat an average distance of 4.9 m from the white screen, with an average visual angle of 21.9° (horizontal) and 16° (vertical). These parameters were expected to maximize affective reactions [26]. Following the instructions, four practice trials, depicting affective and food content, were employed to standardize the participants' understanding and clarify uncertainties (IAPS codes and descriptions: 7021/“Whistle”; 3059/“Mutilation”; 4692/“EroticCouple”; 7451/“Hamburger”). Next, the rating session began. Four additional unannounced practice trials were included so that the participants became familiar with the structure of each trial (5800/“Leaves”; 3130/“Mutilation”; 4085/“EroticFemale”; 7279/“Alcohol”). Each trial consisted of a 4 s display of the number of the upcoming image, 6 s of picture viewing, and 20 s of picture evaluation. The experimenters invited the participants to look at the picture the entire time that it was displayed. Immediately before the rating session, the participants were asked whether (yes/no) they felt hungry. The rating session lasted approximately 30 min and was followed by a 2 min break, after which the participants completed questionnaires (Table 1). The first questionnaire, developed by our research group, assessed basic aspects of the participants' demographics and food/physical activity habits. Further questionnaires probed feelings of social desirability [27], anhedonia [28], and trait food craving [29]. Before leaving, the participants removed their shoes and socks to allow for height measurement (to 0.1 cm) on a Leicester Height Measure stadiometer and weight measurement on an electronic body composition analyzer (Tanita Model 300MA, Chicago, IL) so that body mass index could be estimated.

### Statistical analyses

All statistical analyses were run using Statistica, v.8.0 (Statsoft Inc., Tulsa, OK). Consistent with existing research on emotion [13], we first created the “affective space,” plotting each IAPS and OLAF image according to its average pleasure and arousal ratings and then ensured that pleasure and arousal evaluations were linearly independent. Subsequently, to test that the SAM scales of pleasure, arousal, dominance, and food craving elicited the expected patterns, we ran separate mixed-design analyses of variance (ANOVA), including the participants’ gender and subjective hunger (yes/no, pre-session) as between-subject factors and picture category as a within-subject factor (with 7 levels: pleasant, neutral, unpleasant, sweet high-cal, savory high-cal, low-calorie fruits, and low-calorie vegetables). For all analyses, we set the level of significance at 0.05, employed and reported the Greenhouse-Geisser correction whenever necessary, and informed on effect sizes using the partial η^2^. Afterward, we ran post hoc tests applying the Bonferroni correction to p levels testing multiple differences. To facilitate the interpretation of the data [30], we report the differences between the means with their corresponding Bonferroni-adjusted 95% confidence intervals (CI).

## Results

**Fig 1:**
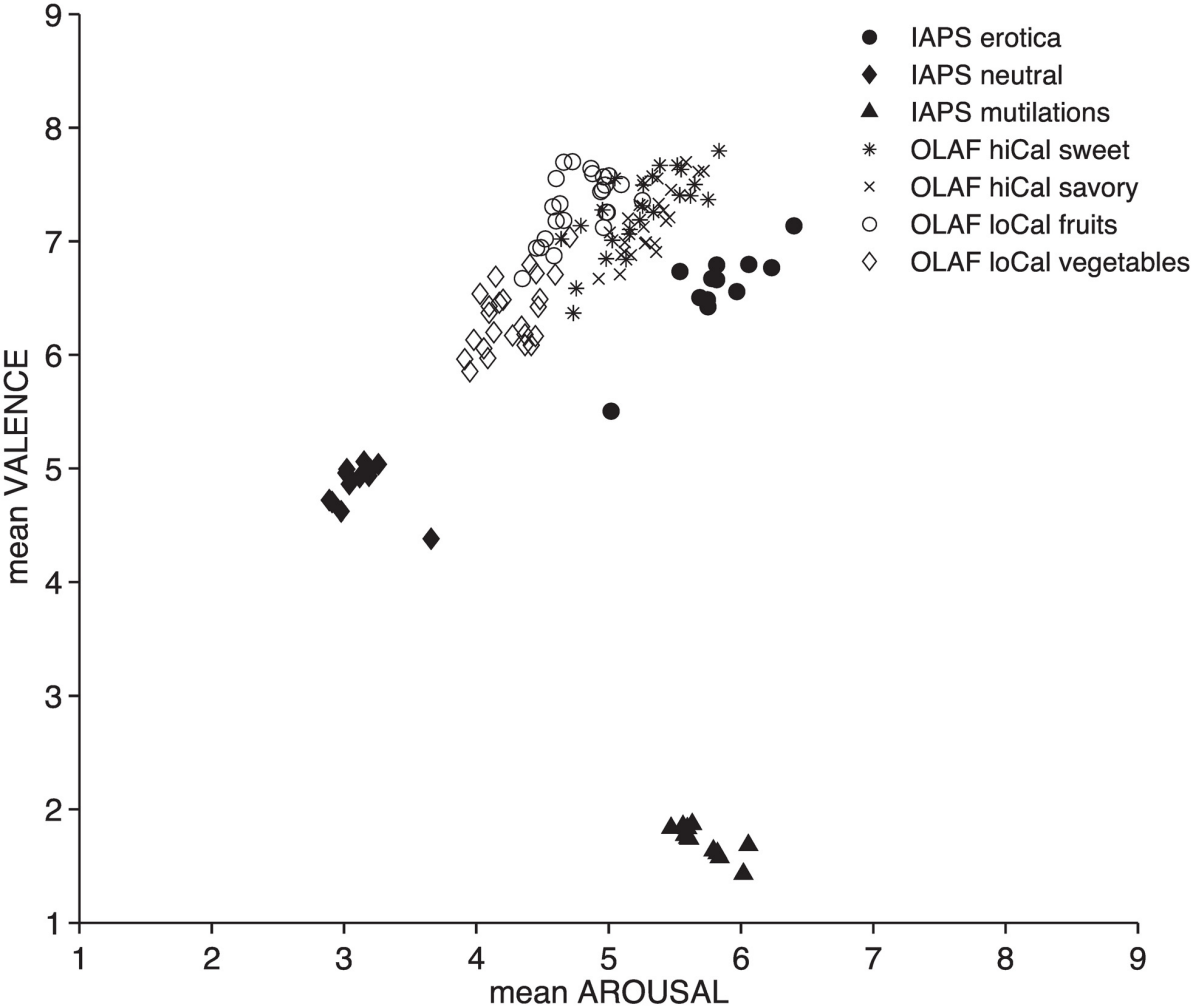
"Affective space." Each dot represents an IAPS or an OLAF image as a function of its mean pleasure (y axis) and arousal (x axis) ratings.

Fig 1 illustrates the “affective space,” that plots each IAPS and OLAF picture as a function of its pleasure and arousal ratings.

Emotional images (solid marks) are on the right, receiving the highest arousal ratings and among the most extreme pleasure ratings (lowest for mutilations, highest for erotica). Neutrals are on the left. As expected, foods prompted very high pleasure ratings but lower arousal ratings, with overall ratings diminishing from high-calorie (crossed symbols) to low-calorie (empty symbols) foods.

Overall, pleasure and arousal ratings were linearly independent (r = 0.044, p = 0.613). Arousal ratings for foods correlated highly with food cravings (r = 0.89, p<0.001).

### Impact of picture category

**Fig 2:**
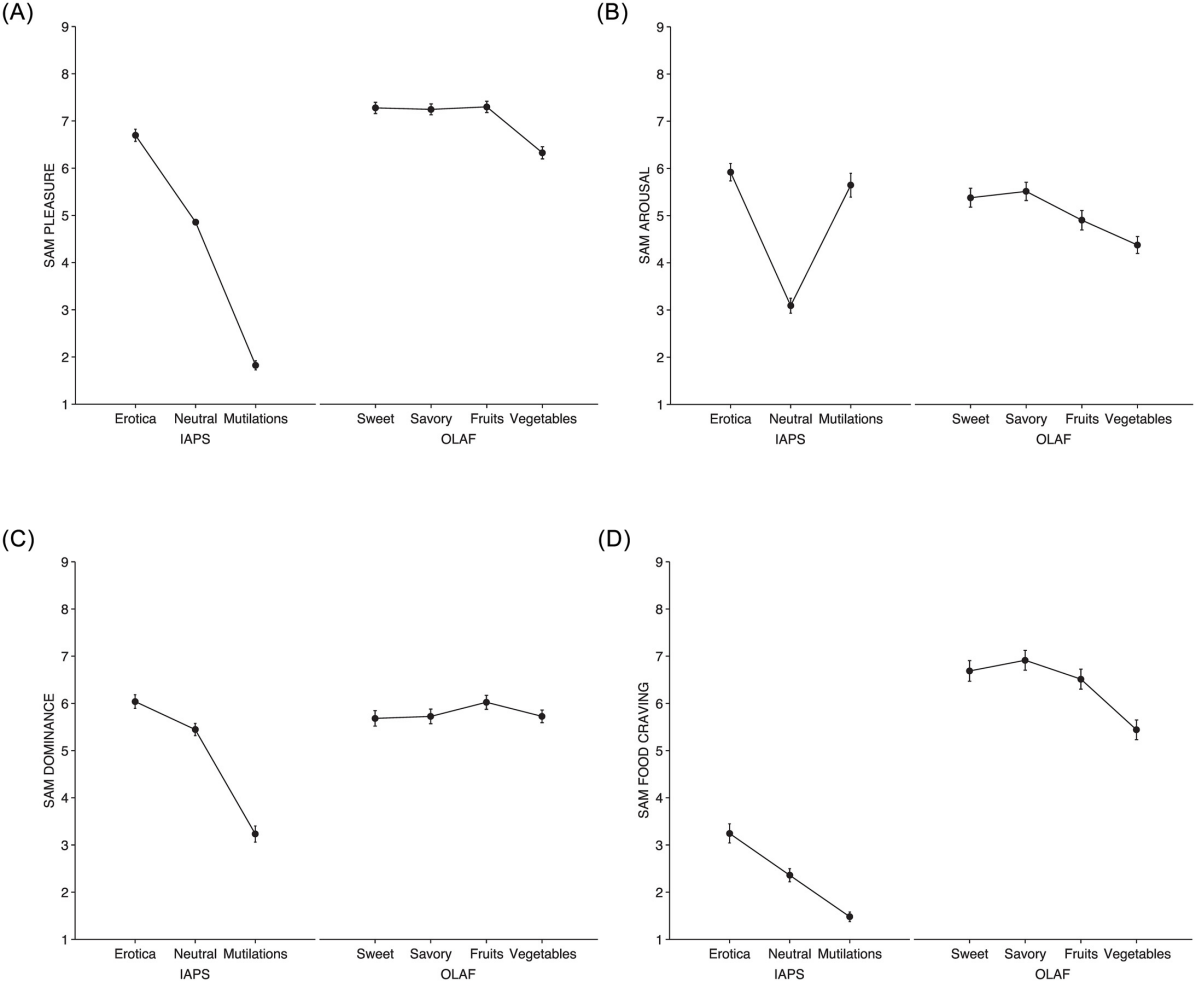
SAM ratings across IAPS and OLAF picture categories for pleasure (2a), arousal (2b), dominance (2c), and food cravings (2d). Erotica, neutral objects, and mutilations prompted the typical patterns in the dimensions of pleasure, arousal, and dominance. For foods, pleasure and arousal jointly provided the more reliable description of the emotional impact of specific food categories, distinguishing more clearly than the other dimensions between sweet and savory high-calorie foods and low-calorie fruits and vegetables. On the contrary, as in our previous work [17], food cravings were less capable of pinpointing the emotional impact of different food categories.

Fig 2 depicts SAM pleasure, arousal, dominance, and food craving ratings across picture categories. Erotica, neutrals, and mutilations are always on the left, foods are on the right, ordered from the high-calorie foods (sweet and savory) to the low-calorie fruits and vegetables. Taken as a whole, when the participants viewed emotional images from the IAPS, classic SAM ratings of pleasure, arousal, and dominance reflected the expected patterns.

#### Pleasure ratings

Fig 2a shows the main effect of picture category on pleasure ratings (F(4.5,1867.7) = 1515.5; p<0.0001; partial η^2^ = 0.78).

Emotional images elicited the typical linear pattern: erotica prompted significantly more pleasure than neutrals (mean difference = 1.74, 95% CI: 1.55–1.92) and neutrals elicited more pleasure than unpleasant contents (mean difference = 3.13, 95% CI: 2.94–3.32).

Among foods, interestingly, images displaying low-calorie fruits and high-calorie foods (either sweet or savory) all prompted the same amount of pleasure (all ps = 1) that was significantly greater than that elicited by images showing erotic contents (95% CI: 0.39–0.85). Vegetables, although significantly lower in pleasure than the other foods, were consistently more pleasing than neutral household objects (mean difference = 1.50, 95% CI: 1.31–1.68). All Bonferroni-adjusted ps<0.0001.

#### Arousal ratings

Fig 2b shows the main effect of picture category on arousal ratings (F(3.3,1393) = 157.7; p<0.0001; partial η^2^ = 0.27).

Erotica and mutilations did not differ (p = 1) in triggering the highest arousal ratings, that were 2.70 greater for erotica (95% CI: 2.42–2.98) and 2.60 greater for mutilations (95% CI: 2.31–2.88) than for neutral contents (all adjusted ps<.0001). Overall, the pattern for arousal ratings of emotional pictures showed the typical quadratic trend.

Sweet and savory high-calorie foods did not differ (p = 1) in causing significantly less activation than erotica (for sweet, 0.58 less, 95% CI: 0.30–0.86; for savory, 0.52 less, 95% CI: 0.23–0.80); however, sugary high-calorie foods triggered 0.45 more arousal than fruits (95% CI: 0.17–0.74). Vegetables, in turn, activated the participants 0.52 less than fruits (95% CI: 0.24–0.80) but a substantial 1.14 more than neutral contents (95% CI: 1.43–0.86. All ps<0.0001).

#### Dominance ratings

Fig 2c shows the main effect of picture category on dominance ratings (F(3.5,1489.9) = 261.9; p<0.0001; partial η^2^ = 0.38).

Looking at progressively more unpleasant content, the participants felt decreasingly dominant (erotica-neutral difference = 0.53, 95% CI: 0.31–0.75; neutral-unpleasant difference = 2.27, 95% CI: 2.0–2.5), showing a pattern consistent with that typically observed in literature on emotions [12].

Erotica prompted the same feelings of control as high-calorie savory and low-calorie foods (all ps>.07); however, among foods, fruits induced the greatest feelings of control, with greater feelings of dominance viewing fruits rather than high-calorie sweet (0.36, 95% CI: 0.14–0.58), high-calorie savory, (0.30, 95% CI: 0.08–0.53), and vegetable foods (0.31, 95% CI: 0.09–0.53).

#### Food craving ratings

Fig 2d shows the main effect of picture category on food craving ratings (F(3.4,1416.5) = 881.5; p<0.0001; partial η^2^ = 0.68).

Food craving ratings while viewing emotional images indicated a linear trend, with more craving for erotica than for neutrals (mean difference = 0.74, 95% CI: 0.46–1.0) and more craving for neutrals than for mutilations (mean difference = 0.69, 95% CI: 0.41–0.96). Thus, the participants extended feelings of craving to non-food-related stimuli, despite explicit SAM instructions that "food craving = 1 when you feel low or no food craving".

High-calorie foods and low-calorie fruits all prompted the same amount of food craving (all ps = 1), which was on average 1.23 units greater than for vegetables (95% CI: 0.83–1.65). In turn, vegetables prompted 2.98 more food craving than neutral objects (95% CI: 2.69–3.25). All adjusted ps<.0001.

### Impact of gender

For food cravings, the significant main effect of gender (F(1,420) = 23.5; p<0.0001; partial η^2^ = 0.05) reflected higher cravings among men than among women (0.77, 95% CI: 0.54–0.99). For the other SAM scales, however, male/female differences were a function of picture category, as indicated by the significant interactions between sex and picture category for pleasure (F(4.4,1867.7) = 24.4; p<0.0001; partial η^2^ = 0.05), arousal (F(3.3,1393) = 5.5; p<0.0001; partial η^2^ = 0.01), dominance (F(3.5,1489.9) = 3.7; p<0.008; partial η^2^ = 0.008), and food craving (F(3.37,1416.5) = 14.1; p<0.0001; partial η^2^ = 0.03). In line with prior literature (Bradley & Lang, 2007), men looking at erotic contents reported an average of 0.85 more pleasure (95% CI: 0.50–1.20), 0.70 more arousal (95% CI: 0.08–1.31), 0.56 more dominance (95% CI: 0.09–1.03), and 1.80 more craving (95% CI: 1.22–2.39) than did women. Moreover, men had higher ratings than women for pleasure when looking at mutilations (0.53, 95% CI: 0.18–0.87) and for food craving when looking at neutrals (0.70, 95% CI: 0.12–1.29).

When viewing images displaying distinct foods, men and women reacted differently: men reported greater cravings in response to savory foods (0.80, 95% CI: 0.21–1.39) and vegetables (0.79, 95% CI: 0.21–1.38), whereas women reported greater pleasure in response to sweet high-calorie foods (0.38, 95% CI: 0.3–0.72) and fruits (0.48, 95% CI: 0.13–0.82).

### Impact of hunger

Our sample was unbalanced for hunger (105 participants reported "yes, I feely hungry", whereas 319 stated "No, I don't feel hungry"), therefore we advise taking with caution the results reported below and exploring further in future investigations the impact of hunger on affective ratings. In general, whenever participants reported feeling hungry, ratings in all categories increased, as indicated by the significant main effects of hunger on pleasure (F(1,420) = 9.4; p<.01; partial η^2^ = 0.02), arousal (F(1,420) = 8.4; p<0.01; partial η^2^ = 0.02), and food craving (F(1,420) = 98.8; p<0.0001; partial η^2^ = 0.19). Furthermore, for arousal ratings, a significant interaction with category (F(3.3,1393) = 7.3; p<.0.0001; partial η^2^ = 0.02) detailed that the impact of hunger was limited to high-calorie foods: participants who reported feeling hungry felt 0.69 more activation looking at sweets (95% CI: 0.002–1.37, p = 0.045) and 0.87 more activation looking at savory foods (95% CI: 0.18–1.56). For food craving, an additional interaction with category (F(3.4,1416.5) = 9.4; p<0.0001; partial η^2^ = 0.02) revealed that hungry participants had on average 1.57 greater cravings in all categories but mutilations (95% CI: 0.55–2.57; all ps<0.0001).

## Discussion

The present work describes the OLAF, the Open Library of Affective Foods, an original set of pictures displaying foods within natural scenes, perceptually matching the features of emotional images, and therefore providing researchers with food stimuli that can be used in affective picture viewing paradigms. Furthermore, each food image from the OLAF is evaluated on different affective dimensions while emotional images from the IAPS are included as non-food controls, therefore providing valid food judgments based on Bartoshuk and colleagues' recommendations [9].

Data reported here, emerged from an adult population, indicate that for emotional images the affective evaluations are consistent with the literature [12,13]. In particular, the impact of picture category and of gender on IAPS ratings of pleasure, arousal, and dominance, together with the lack of association between pleasure and arousal judgments, confirms the reliability of adult OLAF ratings.

Although the affective categories employed as emotional benchmark differed substantially in the adolescent and adult version of the OLAF (in adolescents: adventure scenes, crying babies; in adults: erotica, mutilations), feelings toward high-calorie foods tended to be similar [17]. In adolescents, the feelings of pleasure toward some sweet high-calorie foods were as high as the most pleasant images from the IAPS. Likewise, in adults high-calorie foods, either sweet or savory, prompted greater feelings of pleasure than erotica (this result is probably explained by the slightly greater presence of women in the adult sample, [31]). However, when arousal ratings were considered, in both populations foods were regarded as lower in arousal than the most appetitive IAPS content. Therefore, the inclusion of a wider emotional context highlights that feelings of pleasure (likability) are not enough to describe the emotional impact of food because, in adults, if pleasure alone were considered, fruits would elicit the highest ratings, even higher than those for erotica. It is the inclusion of uncorrelated arousal and pleasure ratings that provides a reliable description of the emotional impact of the images, identifying erotica and mutilations as the “emotionally strongest” categories, followed by high-calorie foods, low-calorie fruits, vegetables, and neutrals. It must nonetheless be kept in mind that given the methodological differences across the adolescent and adult OLAF studies (apart from the emotional anchors, also some less appetizing food images were replaced in the adult version), further investigations are needed for a more reliable comparison of teenage and adult evaluations of food cues.

The patterns of affective ratings in the adult population further emphasize the non-neutrality of vegetables that although lower in pleasure and arousal, nonetheless belong to the appetitive part of the “affective space” and therefore are not an emotionally neutral counterpart to high-calorie foods. This is especially relevant in the context of disordered eating, where vegetables can act as “safety” foods, reducing anxiety yet implicitly confirming the existence of a “threat” in high-calorie foods [32]. As a consequence, affective data suggest that low-calorie foods should not be employed as an “affectively neutral” point of comparison, as some times used within the food cue reactivity paradigm.

Feelings of food craving, as in our previous work on adolescents [17], were not particularly informative. On one hand, they were highly correlated with arousal feelings; on the other hand, they did not distinguish food categories or provide meaningful information for emotional images. Accordingly, we recommend arousal over food craving ratings, at least in the context of emotional processing.

In the introduction, we emphasized the methodological value of adding pleasant, unpleasant, and neutral IAPS pictures, referring particularly to Bartoshuk’s argument [9] that valid evaluations of food likability/pleasure can be made only in comparison with extreme experiences unrelated to food. Likewise, recently, based on psychophysiological studies that identified an “augmented response to food in obesity” using only food and neutral objects, Versace and Schembre [16] called for the inclusion of “adequate high-valenced/high-arousing control stimuli” to draw valid conclusions. Subsequently, Versace and colleagues presented data [33] that illustrate the theoretical richness of investigating emotions in obesity within the affective picture-viewing paradigm: among both lean and obese individuals, some showed the typically moderate brain reactions to food, accompanied by similarly typical augmented physiological reactions to erotica. However, others showed exaggerated brain reactions to food, in turn accompanied by reduced reactions to erotica. Therefore, the inclusion of emotional stimuli allowed the detection of changes in the value of intrinsically appetitive non-food stimuli as a result of the prominence given to food.

The most considerable limitation of the present study lies in its reference to subjective evaluations to predict whether certain visual stimuli can prompt emotional reactions in the human physiology. In particular, although arousal ratings frequently parallel the psychophysiological patterns of human emotion, "there is not always perfect agreement" [34]. Nevertheless, emotional ratings of standardized sets of food images are a considerable step, albeit fallible, toward the identification of food stimuli with the greatest motivational relevance across wide samples of individuals. Moreover, as repeatedly emphasized, the OLAF includes high-arousing affective images from the IAPS as emotional anchors, therefore further guaranteeing the reliability of food picture evaluations [9]. Additional shortcomings of the OLAF are its limited size (and subsequent ability to represent the enormous diversity of foods in daily life) and the doubts over its likability in non-Spanish cultures. It should be noted, however, that the preference for local familiar foods [35] is to some extent unavoidable, given its evolutionary rationale of exposing individuals to safer, known foods (as compared to unknown, riskier food items). On the other hand, the current globalized food system partially circumvents the preference for local foods.

In summary, ratings for affective images were in line with our hypotheses, confirming the reliability of the OLAF images and ratings. We believe that the concurrent inclusion of food and emotional pictures can methodologically and theoretically broaden and strengthen research on emotions in obesity.
